# Induction of HLA-A2 restricted CD8 T cell responses against ApoB100 peptides does not affect atherosclerosis in a humanized mouse model

**DOI:** 10.1038/s41598-019-53642-z

**Published:** 2019-11-22

**Authors:** Frank H. Schaftenaar, Jacob Amersfoort, Hidde Douna, Mara J. Kröner, Amanda C. Foks, Ilze Bot, Bram A. Slütter, Gijs H. M. van Puijvelde, Jan W. Drijfhout, Johan Kuiper

**Affiliations:** 1 Division of BioTherapeutics, Leiden Academic Centre for Drug Research, Leiden, The Netherlands; 2 Department of Immunohematology and Blood Transfusion, Leiden University Medical Center, Leiden, The Netherlands

**Keywords:** Lymphocyte activation, Peptide vaccines, Cardiovascular diseases

## Abstract

Cardiovascular diseases form the most common cause of death worldwide, with atherosclerosis as main etiology. Atherosclerosis is marked by cholesterol rich lipoprotein deposition in the artery wall, evoking a pathogenic immune response. Characteristic for the disease is the pathogenic accumulation of macrophages in the atherosclerotic lesion, which become foam cells after ingestion of large quantities of lipoproteins. We hypothesized that, by inducing a CD8 T cell response towards lipoprotein derived apolipoprotein-B100 (ApoB100), lesional macrophages, that are likely to cross-present lipoprotein constituents, can specifically be eliminated. Based on *in silico* models for protein processing and MHC-I binding, 6 putative CD8 T cell epitopes derived from ApoB100 were synthesized. HLA-A2 binding was confirmed for all peptides by T2 cell binding assays and recall responses after vaccination with the peptides proved that 5 of 6 peptides could induce CD8 T cell responses. Induction of ApoB100 specific CD8 T cells did not impact plaque size and cellular composition in HLA-A2 and human ApoB100 transgenic LDLr^−/−^ mice. No recall response could be detected in cultures of cells isolated from the aortic arch, which were observed in cell cultures of splenocytes and mesenteric lymph nodes, suggesting that the atherosclerotic environment impairs CD8 T cell activation.

## Introduction

Cardiovascular disease is the most common cause of death in the western world with atherosclerosis as the most common etiology^1^. Atherosclerosis is characterized by lipid deposition in the intima of medium to large-sized arteries, evoking immune infiltration in the vessel wall and inflammation. Among the immune cells attracted to the atherosclerotic lesions are CD8 T cells^2^. CD8 T cells in atherosclerotic lesions appear highly activated^3^ suggesting a pathogenic role for CD8 T cells atherosclerosis. Recent studies indeed support the notion of an overall pro-atherogenic role of CD8 T cells in atherosclerosis through the secretion of pro-inflammatory cytokines^4–6^. Nonetheless, atheroprotective effects of CD8 T-cells in the lesion have been reported as well^7^.

These conflicting reports may result from the fact that CD8 T cells form a heterogeneous population consisting of different subsets and cells recognizing different antigens. The largest CD8 T cell subset is the cytotoxic CD8 T cell or cytotoxic lymphocyte (CTL)^8^. The primary function of CTLs is to protect the host from intracellular pathogens^9^ and tumors^10^. Specific recognition of a target cell is established through positive interaction between the T cell receptor of the CTL, which is variable between individual CD8 T cells, and MHC-I complexed with a particular antigen derived peptide on the target cell. MHC-I/peptide complex recognition results in TNF-α and IFN-γ production by CTLs and leads to FAS ligand or perforin and granzyme B mediated cell death of the target cell^11^. The specificity of the TCR heavily impacts its pathogenicity, as depending on target antigen, vaccination induced CTLs can be atheroprotective^12–15^ or atherogenic^16^. Induction of CTL reactivity towards vascular cells like smooth muscle cells, enhanced vessel inflammation and atherosclerosis^16^. As suppressing apoptosis of macrophages was found to enhance atherosclerosis^17,18^ and the absence of MHCI molecules aggravates atherosclerosis^19^ we hypothesize that CD8 T cell mediated killing of macrophages is an essential process controlling progression of atherosclerosis. Intra-lesional TLR activation^20^ and presence of apoptotic bodies^21^, both potent inducers of cross presentation^22,23^, are likely to induce cross presentation of plaque antigens by lesion macrophages. Presumable cross-presentation of LDL derived apolipoprotein-B100 (ApoB100) epitopes on MHC-I by lesional phagocytes, suggests that inducing ApoB100 specific CD8 T cells could lead to killing of lesional macrophages and reduce atherosclerosis.

Since only a very small fraction of peptides binds to MHC-I^24^, we set out to identify CD8 T cell epitopes in ApoB100 to test this hypothesis. With *in silico* prediction models for HLA binding and antigen processing, human HLA-A2 restricted epitopes derived from human ApoB100 were predicted for translational relevancy. 6 ApoB100 derived peptides were selected and synthesized and binding of all peptides to HLA-A2 was confirmed with HLA-A2 assays in T2 cells^25^. Thereafter we performed vaccination studies using these peptides, inducing substantial levels of peptide specific memory CD8 T cells in HLA-A2 and human ApoB100 transgenic LDLr^−/−^ mice. Although ApoB100 specific CTLs were induced by ApoB100 peptide vaccination, these CD8 T cells did not change cellular plaque composition, plaque collagen content, and plaque size, indicating that induction of ApoB100 specific CD8 T cells does not affect atherosclerosis.

## Results

### Predicted HLA-A2 restricted epitopes stabilize HLA-A2 and induce peptide specific CD8 T cell responses after DC vaccination

**Figure 1:**
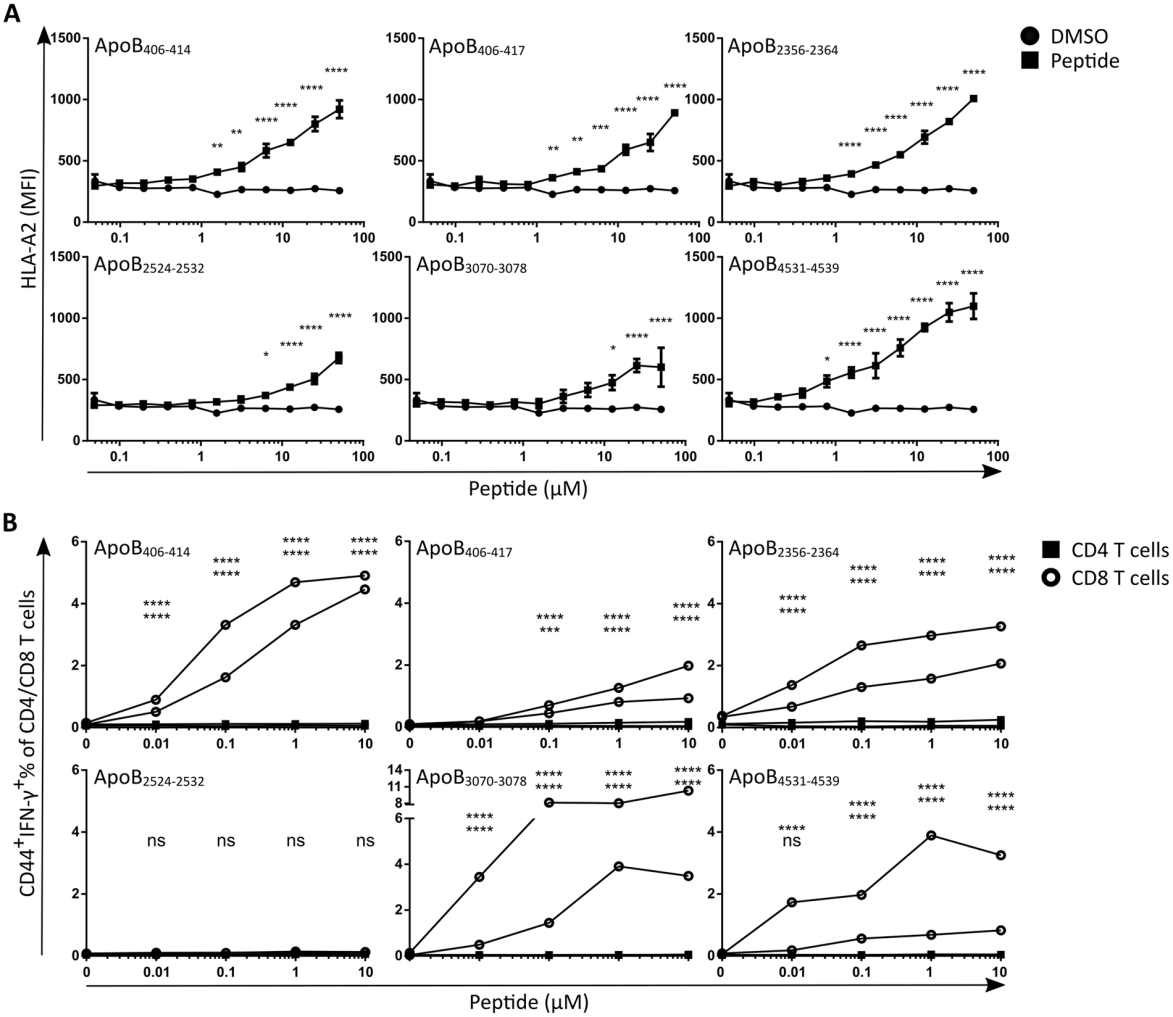
Predicted HLA-A2 restricted epitopes stabilize HLA-A2 and induces peptide specific CD8 T cell responses after DC vaccination. (**A**) Binding of in silico predicted CD8 T cell epitopes was assessed using T2 cells. T2 cell cultures (n = 3/peptide) were incubated overnight with a range of peptide concentrations or vehicle control (DMSO) and assessed for HLA-A2 expression by flow cytometry. (**B**) Next we assessed whether these ApoB100 derived peptides could induce an HLA-A2 restricted CD8 T cell response. HLA-A2tg mice (n = 2 per peptide), not expressing human ApoB100, were vaccinated with 2*10^6^ HLA-A2tg bone marrow derived DCs pulsed with a single peptide (30 μM). A week after vaccination splenocytes were *ex vivo* incubated with the peptide against they were vaccinated. Graphs of peptide specific T cell responses a measured by flow cytometry through gating for CD44 and IFN-γ double positive T cells. Statistical analysis of A was performed with 2-way ANOVA and Bonferroni posttest, displayed as mean with SEM. For B, samples stimulated with peptide were compared to the unstimulated control of the same animal with contingency chi-square tests and Bonferroni posttest (corrected for 96 pairwise comparisons), individual samples are plotted. *p < 0.05, **p < 0.01, ***p < 0.001, ****p < 0.0001.

To target CD8 T cells towards plaque macrophages which are likely to cross-present plaque derived antigens, we predicted putative HLA-A2 restricted CD8 T cell epitopes in human ApoB100 using in silico models for immunoproteasomal processing and TAP binding^26–28^ and HLA-A2 binding models^28–35^. We synthesized 6 peptides with the highest putative HLA-A2 binding and processing score (Table 1). To establish the binding of the ApoB100 peptides to HLA-A2 *in vitro*, T2 cells were incubated overnight with ascending amounts of ApoB100 peptides and thereafter the expression of HLA-A2 on the T2 cells with flow cytometry was assessed (Supplementary Fig. S1). Because T2 cells are deficient for TAP1 and TAP2 expression, cytosolic peptide transport into the ER is reduced in T2 cells, reducing endogenous peptide loading onto MHC-I in the ER. MHC-I not complexed with a peptide is inefficiently transported to the cell membrane and is less stable, therefore leading to lowered expression of HLA-A2 on the cell membrane of T2 cells. Exogenous addition of peptides, forming MHC-I/peptide complexes on the cell surface, enhances MHC-I stability on the plasma membrane which increases expression of HLA-A2 on the T2 cell surface^25^. All 6 ApoB100 peptides increased HLA-A2 expression in a concentration dependent manner (Fig. 1A), confirming that these peptides bind to HLA-A2.

**Table 1:** ApoB100 derived CD8 T cell epitope processing and HLA-A2 binding prediction.

Peptide	Amino Acid Sequence	NetMHCpan (IC50)	Concensus Percentile	Processing Score	Final Score
ApoB_2356–2364_	VLMDKLVEL	2.95	0.2	2.32	1.85
ApoB_406–414_	LLIDVVTYL	2.48	0.2	1.9	1.5
ApoB_406–417_	LLIDVVTYLVAL	3.96	0.2	1.89	1.3
ApoB_2524–2532_	YQMDIQQEL	5.19	0.5	1.9	1.19
ApoB_3070–3078_	FLNNYALFL	4.75	0.3	1.86	1.18
ApoB_4531–4539_	FLIYITELL	4.76	0.4	1.8	1.12

To test the immunogenicity of the peptides we vaccinated HLA-A2 transgenic animals (HHD mice)^36^ with HHD bone marrow derived DCs (2*10^6^ cells), which were overnight stimulated with LPS (100 ng/ml) and pulsed with a single peptide (10 µM). One week after vaccination, we assessed the induction of peptide-specific CTLs through flow cytometric measurement of IFN-γ positive antigen experienced (CD44^+^) CD4 and CD8 T cells (Supplementary Fig. S1), in splenocyte cultures which were incubated for 4 h with escalating concentrations of the ApoB100 peptide against which the particular animal was vaccinated, and Brefeldin A. For all peptides, except for ApoB_2524–2532_, the percentage of IFN-y positive antigen experienced CD8 T cells was increased over no peptide control, while the vaccinations did not affect CD4 T cell IFN-y production (Fig. 1B). This indicates that 5 of 6 peptides can induce HLA-A2 restricted CD8 T cell responses *in vivo*.

### Peptide vaccination induces peptide responsive CD8 T cells in spleen and mediastinal lymph nodes but not in aorta

A suitable experimental model to assess the role of human ApoB100 specific T cells in the context of atherosclerosis was generated by crossbreeding HHD mice, transgenic for HLA-A2 and deficient in expression of murine MHC-I molecules^36^, with HuBL mice, expressing human ApoB100 and deficient for the LDLr^37,38^. HLA-A2 and human ApoB100 transgenic mice, which were LDLr deficient to allow atherosclerosis development, and either with normal expression (HuBL-A2^m+^) or devoid of murine MHC-I expression (HuBL-A2^m−^) were generated. Because immunization with the ApoB100 peptides in both mouse strains yielded similar immunological results and effect on plaque parameters in the atherosclerosis studies, mainly the results obtained with female HuBL-A2^m−^ mice are shown with some complementary data obtained in male HuBL-A2^m+^ mice.

**Figure 2:**
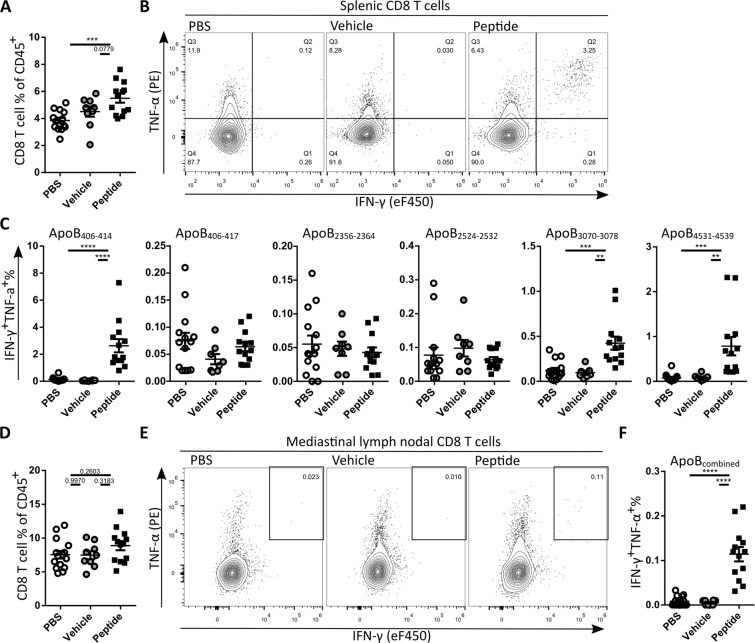
Peptide vaccination induces peptide responsive CD8 T cells. (**A**) Quantification of splenic CD8 T cell percentage. (**B**) Representative flow cytometry plots of splenic CD8 T cell activation by ApoB_406–414_ in the different treatment groups. (**C**) Quantification of IFN-γ and TNF-α double positive cells % from CD8 T cells. (**D**) Quantification of CD8 T cell percentage after *ex vivo* peptide stimulation mediastinal lymph nodes. (**E**) Representative flow cytometry plots of mediastinal lymph node CD8 T cell activation and (**F**) quantification of the IFN-γ and TNF-α double positive cell % from CD8 T cells after combined peptide stimulation. Statistical analysis was performed with 1-way ANOVA and Tukey’s multiple comparisons test. Depicted as mean with SEM, **p < 0.01, ***p < 0.001, ****p < 0.0001.

HuBL-A2^m−^ mice received western type diet for 9 weeks starting from 15 weeks of age. Mice of the treatment group were i.v. vaccinated with HHD DCs (2*10^6^ cells) pulsed with a mixture of the 6 ApoB100 derived peptides (10 μM/peptide) at the start of western type diet. Mice were i.v. boosted a week later with peptides (30 μM/peptide) adjuvanted with anti-CD40 (50 μg) and poly(I:C) (50 μg), previously shown to induce long lasting high levels of antigen-specific memory CD8 T cells^39–41^. Control animals received unpulsed control DCs (2*10^6^ cells) at WTD initiation and a week later adjuvant without peptides (vehicle group), or received two PBS injections (PBS group). At the end of the experiment we determined whether vaccination induced peptide specific CD8 T cells. To that end, CD8 T cell recall responses were assessed with flow cytometry in cell cultures of the spleen (2*10^6^ cells) and mediastinal lymph nodes (1*10^6^ cells), which drain from the atherosclerosis prone aortic arch, incubated for 4 h with the ApoB100 derived peptides (10 μM/peptide) and Brefeldin A. The CD8 T cell percentage was significantly higher in the peptide-treated group compared to the PBS group and trended to be higher compared to the unpulsed DC/vehicle group in spleen (Fig. 2A) and blood (Supplementary Fig. S2) but not in mediastinal lymph nodes (Fig. 2D). Incubation of splenocytes with ApoB_406–414_, ApoB_3070–3078_ or ApoB_4531–4539_ enhanced the percentage of IFN-γ and TNF-α double positive CD8 T cells in the splenocytes cultures of the peptide-treated group, indicative of successful long-term induction of memory CD8 T cells for these peptides (Fig. 2B,C). In cell cultures from the mediastinal lymph nodes, incubated with a combination of all peptides, also a higher percentage of IFN-γ^+^ and TNF-α^+^ double positive CD8 T cells was observed in the peptide vaccinated group (Fig. 2E,F), although at a much lower percentage than in the spleen. In circulation, a strong increase in effector memory CD8 T cells and a reduction in naïve CD8 T cells were observed in the peptide-treated group compared to both control groups (Supplementary Fig. S2), suggesting that vaccination also resulted in peptide specific CD8 T-cells in circulation.

### Vaccination with ApoB100 derived CD8 epitopes does not affect atherosclerotic lesion size and composition

**Figure 3:**
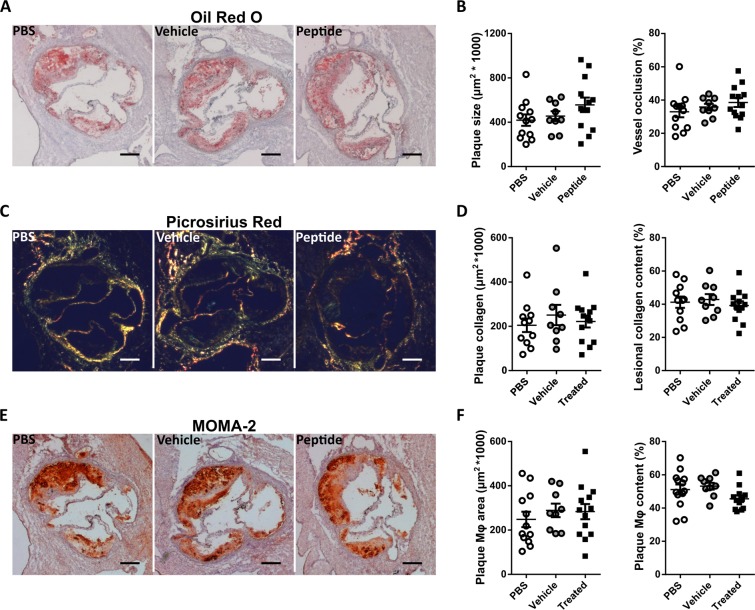
Vaccination with ApoB100 derived CD8 epitopes does not affect lesion size and composition. (**A**) Representative microscopic images of ORO staining of aortic root tissue sections. (**B**) Quantification of average plaque size and average vessel occlusion. (**C**) Plaque stability was assessed by collagen content determination through analysis of Sirius red stained tissue slides. Representative microscopic images of aortic root tissue sections stained with Sirius red. (**D**) Quantification of collagen content of atherosclerotic lesions in the aortic root. (**E**) Representative microscopic images of tissue slides stained with MOMA-2 staining (**F**) and quantification of MOMA-2 surface area and plaque content. Statistical analysis was performed with 1-way ANOVA and Tukey’s multiple comparisons test. Plotted as mean with SEM.

To assess the effect of ApoB100 specific CD8 T cell induction on atherosclerotic lesion development, we stained neutral lipids in aortic root sections with ORO (Fig. 3A), and quantified plaque size (control 4.18 ± 1.79*10^5^, vehicle 4.55 ± 1.32*10^5^, treated 5.57 ± 2.35*10^5^) and vessel occlusion (Fig. 3B). To assess the stability of the plaques we stained aortic root sections with Masson’s Trichrome (Fig. 3C), and quantified the absolute area of plaque collagen and collagen content of the plaques (Fig. 3D). Finally, with immunohistochemical staining with MOMA-2 antibody (Fig. 3E), we quantified the absolute macrophage area and macrophage content of aortic root atherosclerotic plaques (Fig. 3F). None of the mentioned plaque parameters were significantly changed by induction of ApoB100 specific CD8 T cells. Also, cholesterol levels were not influenced by ApoB100 peptide vaccination (Supplementary Fig. S3).

### ApoB100 peptide vaccination enhances CD8 T cell content but does not affect other immune cell populations in the aorta

Because the induced ApoB100 peptide specific CD8 T cells were hypothesized to reduce atherosclerosis through killing of plaque phagocytes cross-presenting the ApoB100 peptides, we assessed peptide reactivity and phenotype of CD8 T cells in the aortic arch, and the general immune content of the aortic arch.

**Figure 4:**
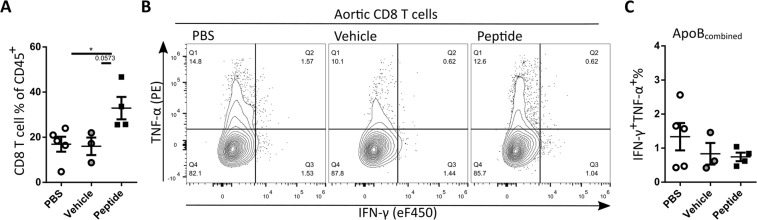
Vaccination with ApoB100 peptides does enhance CD8 T cell levels but not reactivity towards ApoB100 peptides in the aortic root (**A**) Quantification of CD8 T cell percentage in aortic cultures. (**B**) Representative flow cytometry plots of aortic CD8 T cell activation and (**C**) quantification of IFN-γ and TNF-α double positive cell % from CD8 T cells after restimulation with a mix of all peptides. Statistical analysis was performed with 1-way ANOVA and Tukey’s multiple comparisons test. Depicted as mean with SEM, *P < 0.05.

To assess reactivity of CD8 T cells to the ApoB peptides, aortas from three mice from the same treatment group were pooled, digested, and cells were isolated and cultured for 4 h with all peptides combined (10 μM/peptide) and Brefeldin A, after which cells were assessed with flow cytometry (gating, Supplementary Fig. S4). The total percentage of CD8 T cells was significantly increased in the peptide treated group over PBS treated, and a trend towards increase (p = 0.0573) was observed compared to the vehicle treated (Fig. 4A). In contrast to the observed recall responses in the spleens and mediastinal lymph nodes from mice vaccinated with the ApoB100 peptides, we did not observe a recall response in the cell cultures from the aortic arch of the mice vaccinated with ApoB100 peptides (Fig. 4B,C).

In the atherosclerosis study using HuBL-A2^m+^ mice, the aortic CD8 T cell level, their maturation and activation was assessed with flow cytometry directly after isolation. In this study, a significant increase in CD8 T cell levels was found in ApoB peptide treated mice compared to vehicle treated mice, suggesting that migration of ApoB peptide specific T cells into the plaque occurred. We did not observe phenotypical differences in the aortic CD8 T cell population between vehicle and peptide treated animals (Supplementary Fig. S5). Besides an increase in CD8 T cells we did not detect significant changes in multiple myeloid and lymphoid cell populations in the aortic arch, shown for the aortic arch cultures of HuBL-A2^m−^ mice (Supplementary Fig. S4), and myeloid and lymphoid cell populations directly after isolation from the aortic arch from HuBL-A2^m+^ mice (Supplementary Fig. S6).

As CD8 T cells require cell-cell contact for killing of target cells^42^, we also assessed whether CD8 T cells co-localized with macrophages, the most prominent APC population in the atherosclerotic lesion, in aortic root sections of female HuBL-A2^m−^ mice. CD8 T cells were predominantly present in the cap structures of the atherosclerotic lesion possibly interacting with macrophages in the outer layers of the macrophage-rich area, and in addition we observed CD8 T cells in the adventitia. Macrophages were mostly present in the deeper layers of the plaque but in some tissue sections we also do observe CD8 T cells deeper in the plaque (Supplementary Fig. S7).

Thus, our data suggest that ApoB100 specific CD8 T cells do not have a major impact on the size and composition of the atherosclerotic lesion.

## Discussion

The role of CD8 T cells in atherosclerosis is complex, with different roles for different CD8 T cell subsets and CD8 T cells with different antigen specificities. Recent studies suggest an overall pathogenic role for CTLs through secretion of pro-inflammatory cytokines^4,5^, which seems to correlate with observations of increased activated and cytokine producing CD8 T cells in peripheral blood of patients with coronary artery disease^43–45^. However, depending on target antigen, atherogenic and also atheroprotective^12–14^ antigen specific CD8 T cells responses have been reported. Because the suppression of macrophage apoptosis enhanced atherosclerosis^17,18^ and the absence of MHCI molecules aggravates atherosclerosis^19^, we reasoned that promoting CD8 T cell mediated killing of macrophages could act atheroprotective. As the inflammatory plaque environment is likely to promote cross-presentation of plaque antigens by lesional macrophages^20–23^, we hypothesized that we could specifically target CD8 T cells to kill lesional macrophages through vaccination with ApoB100 derived CD8 T cell epitopes, which was proposed to be the atheroprotective mechanism behind vaccination with ApoB100 derived peptide p210^13,15^.

To be applicable for vaccination in men, the CD8 epitopes have to be capable of binding human MHC-I. Therefore we predicted human HLA-A2 binding affinity and immunoproteasomal processing of peptide sequences present within ApoB100 with in silico prediction tools^26–35^. In line with a high accuracy of MHC-I binding models at predict MHC binders and non-binders^46^, all 6 predicted peptides were found to bind HLA-A2 in T2 cell binding assays. Subsequently we assessed the immunogenicity of the peptides in HLA-A2 transgenic HHD mice^36^. Vaccination of HHD mice with HHD DCs pulsed with a single ApoB100 derived peptide, led to robust peptide specific recall responses a week after vaccination for all peptides except for ApoB_2524–2532_, confirming immunogenicity for all ApoB100 derived peptides except ApoB_2524–2532_. Through utilization of mice and DCs without murine MHC-I expression, the observed CD8 T cell responses had to be restricted to HLA-A2^36^.

The peptides used in this study were not previously assessed for their potential to modulate atherosclerosis. However, antibodies in the blood directed against LQWLKRVHANP**LLIDVVTYLVAL**IPEPS, and **FLIYITELL**KKLQSTTVMNPYMKLAPGELT, containing the ApoB_406–417_ (LLIDVVTYLVAL), ApoB_406–414_ (LLIDVVTYL) and ApoB_4531–4539_ (FLIYITELL) peptides, were screened as a potential biomarker for acute myocardial infarction^47^. Antibodyies against LQWLKRVHANP**LLIDVVTYLVAL**IPEPS and **FLIYITELL**KKLQSTTVMNPYMKLAPGELT were found in blood of patients suffering from acute myocardial infarction and individuals in the control group, and did not correlate with acute myocardial infarction^47^. Our vaccination approach is highly unlikely to have induced high antibody levels against the peptides, as priming occurred with peptide-pulsed MHC-I loaded DCs, followed by only a single booster with soluble peptide. Furthermore, a library screening of ApoB100 peptides for their capacity to induce T cell proliferation revealed that a peptide with the amino acid sequence HT**FLIYITELL**KKLQSTTVM, containing the ApoB_4531–4539_ (FLIYITELL) peptide that we used, could enhance proliferation of spleen derived T cells^48^.

To induce long lasting peptide specific CTLs in the atherosclerosis studies, HuBL-A2^m+/−^ mice were primed with peptide pulsed HHD DCs, and then boosted after a week with peptide adjuvanted with an agonistic CD40 antibody and poly(I:C). In experimental cancer models this vaccination approach induces CTLs that are effective in penetrating and killing tumor cells, indicating that this vaccination approach yields migratory and functional CTLs^39–41^. Moreover multivalent vaccination enhanced the anti-tumor effect of this immunization approach^41^. For ApoB_406–414_, ApoB_3070–3078_, and ApoB_4531–4539_, robust recall responses were detected in the spleens of ApoB100 peptide vaccinated HuBL-A2^m+/−^ mice, indicating successful vaccination. The vast majority of the ApoB100 specific CD8 T-cells made more than one cytokine (IFN-γ and TNF-α) suggesting they are functional CTLs^49^. Interestingly no CD8 T cell recall response could be detected for ApoB_406–417_ and ApoB_2356–2364_, suggesting thymic negative selection or peripheral tolerance induction towards ApoB_406–417_ and ApoB_2356–2364_ in the human ApoB100 expressing HuBL-A2^m−/+^ mice. Besides enhanced levels of ApoB100 peptide specific CD8 T cells in the spleen, we observed peptide specific CD8 T cells in the mediastinal lymph nodes, more effector CD8 T cells in the circulation and increased CD8 T cell levels in the aorta of ApoB100 vaccinated mice.

Despite induction of this relatively large antigen specific CD8 T cell response compared to studies in which p210 coupled to cationic BSA (p210-cBSA) was reported to reduce atherosclerosis through CD8 T cell induction^13,15^, we did not observe an atheroprotective effect of ApoB100 specific CD8 T cell induction. Although *in silico* prediction models suggest that p210 does not harbor CD8 T cell epitopes (murine H2Db and H2Kb MHC-I alleles), CD8 T cell involvement in the atheroprotective effect of p210-cBSA was shown by the transfer of atheroprotection by CD8 T cell transfer from p210-cBSA vaccinated donors to recipients, not observed in recipients from CD8 T cells of vehicle treated donors^13^. In our hands however, various vaccination protocols including vaccination of alum adjuvanted p210 coupled to PADRE (Pan-DR epitope), resulted in antibodies against p210 but did not affect the CD8 T cell population in human ApoB100 transgenic LDLr^−/−^ (unpublished results). As FITC-p210 was more effectively taken up by DCs than unconjugated FITC^13^, this suggests that p210 possesses adjuvant properties. The LDLr binding sites of ApoB100, including p210, were also used in a construct with CD8 T cell epitope SIINFEKL to promote cross-presentation of SIINFEKL and induce SIINFEKL specific CD8 T cell activation^50^. As the antigen specificity of CD8 T cells after cBSA-p210 immunization was not assessed, this could imply that p210 acted as adjuvant for cBSA and enhanced uptake and (cross-) presentation of cationic BSA. As immunization with cationic BSA was previously reported to reduce atherosclerosis^51^, enhancing the immune response against cBSA could underlie the atheroprotective effect of cBSA-p210 vaccination.

As we observed strong recall responses towards 3 ApoB100 derived peptides, it is unlikely that the quality and quantity of the induced CD8 T cell response was insufficient to modulate atherosclerosis. As we observed a differential localization pattern of CD8 T cells and macrophages in the plaque it is possible that APCs and CD8 T cells not sufficiently co-localize to impact atherosclerosis. Alternatively it is possible that, although TLR induction and engulfment of apoptotic bodies induce cross presentation^23,52^, other lesional factors could have hampered cross-presentation, such as oxidized lipids which cause disturbance of lipid bodies^53^ which were found essential for cross presentation in DCs^54,55^. The observation that profoundly reduced cross priming capacity in batf3^−/−^ chimeras does not affect atherosclerosis, suggests that cross presentation is not important in atherosclerosis^56^. In our restimulation assays however, lack of cross presentation and lack of co-localization is circumvented by exogenous addition of peptide which externally binds to MHC-I. Despite enhanced CD8 T cell levels in the aortas of ApoB peptide treated mice, we did not observe peptide responsiveness in the cultures with cells derived from the aortic root, suggesting that the atherosclerotic environment reduces the ability of CD8 T cells to respond to TCR stimuli. It is possible that this lack in responsiveness is CD8 T cell intrinsic, e.g. due to chronic antigen exposure in the plaque leading to CD8 T cell exhaustion^57,58^. In line with CD8 T cell exhaustion in atherosclerosis, upregulation of the co-inhibitory PD-1 expression was observed in atherosclerosis patients^59^. On the other hand, plaque cells could inhibit CD8 T cell activation, e.g. PD-L1 was found upregulated on macrophages in human lesions (Watanabe *et al*., 2017), which could provide a co-inhibitory signal to plaque CD8 T cells. The potential of plaque APCs to induce T cell activation is however largely unidentified, but would be very interesting to assess. Moreover it was recently shown that CD8 T-cells in the atherosclerotic lesion significantly downregulate cytokine production as a result of local adenosine signaling^60^. Therefore, it is possible that impaired antigen specific activation of plaque CD8 T cells, could have rendered induction of ApoB100 specific CD8 T cells ineffective.

MHC-I epitope elution from plaque material and single cell TCR sequencing of lesional CD8 T cells could greatly enhance understanding of CTL biology in atherosclerosis, especially since new bioinformatics avenues are opening up that allow linking of TCR sequences to antigens^61–63^. Identification of these antigens could help unravel atherogenic and atheroprotective CTL mediated immune responses, uncovering treatment options of atherosclerosis through vaccination of antigen specific CTLs.

In conclusion, here we assessed the effect of vaccination with ApoB100 derived CD8 T cell epitopes on atherosclerosis development. We could generate and maintain a very robust CD8 T cell response against 3 epitopes over 8 weeks, however in contrast to other studies which report CD8 T cell mediated protection from atherosclerosis resulting from vaccination with ApoB100 derived peptides^13,15^, boosting CD8 T cell immunity towards ApoB100 did not reduce atherosclerosis in our hands.

## Materials and Methods

### In silico HLA-A2 restricted human ApoB100 CD8 T cell epitope prediction

With use of the immune epitope database and analysis resource (iedb.org), putative human ApoB100 derived HLA-A2 restricted CD8 T cell epitopes were predicted. First top 1% predicted binders were selected using NetMHCpan^29^ which was reported to be the best prediction model for HLA-A2^64^. Thereafter the Proteasomal cleavage/TAP transport/MHC class I combined predictor was used to rank the remaining peptides based on proteasomal cleavage and TAP transport^26,27^ and a consensus binding prediction^28^ combining Artificial neural network (ANN)^31–34^, Stabilized matrix method (SMM)^30^, and Scoring Matrices derived from Combinatorial Peptide Libraries (CombLib) models^35^. The 6 top peptides were synthesized (IHB, Leiden University Medical Centre).

### HLA-A2 binding assay

10 mM peptide stocks were prepared in DMSO because of poor water solubility of the peptides and further diluted into complete IMDM. T2 cells, a kind gift from dr. Heemskerk (Leiden University Medical Centre, Leiden) were incubated with peptide concentrations ranging from 0.01–50 μM overnight and 3 μg/ml β-2 microglobulin (Sigma-Aldrich), and HLA-A2 expression was assessed with flow cytometry.

### Animals

All animal work was approved by the Leiden University Animal Ethics Committee and the animal experiments were performed conform the guidelines from Directive 2010/63/EU of the European Parliament on the protection of animals used for scientific purposes. Human HLA-A2 transgenic (HHD), H-2D^b−/−^ β2m^−/−^ C57BL/6 mice^36^, a kind gift from dr. Lemonnier (Institut Pasteur, Paris), were crossbred with human ApoB100 transgenic LDLr^−/−^ C57BL/6 mice^37,38^, a kind gift from dr. Nilsson (Lund University, Lund), to generate human ApoB100 and HLA-A2 transgenic H-2D^b−/−^ β2m^−/−^ LDLr^−/−^ (without murine MHC-I expression, HuBL-A2^m−^) and hApoB100 and HLA-A2 transgenic H-2D^b+/+−^ β2m^+/+−^ LDLr^−/−^ mice (with murine MHC-I expression, HuBL-A2^m+^). Expression of ApoB100 in blood plasma, was determined with the ApoB100 ELISA developer kit (Mabtech) according to manufacturer’s protocol. Expression of HLA-A2, H2Db and H2Kb on peripheral white blood cells was assessed with flow cytometry using HLA-A2-APC (BB7.2, Biolegend), H2Db-FITC (28-14-8, ThermoFisher) and H2Kb-BV421 (AF6-88.5, BD Biosciences) antibodies respectively. Blood for fenotyping purposes was obtained by lateral tail cut and collected in EDTA coated capillary tubes (Microvette®, Sarstedt). LDLr deficiency was assessed with PCR (Primers: forward-common; CAGTGCTCCTCATCTGACTTG, reverse-WT; CATCTCCCCGCAGTTTGTGT, reverse-KO; CGCCTTCTTGACGAGTTCTTCTG). All mice were kept in individual ventilated cages with aspen bedding, in groups of 2–4 mice per cage and were fed a regular chow diet or a ‘Western-type’ diet (WTD) containing 0.25% cholesterol and 15% cocoa butter (Special Diet Services, Witham, Essex, UK). All mice used in experiments were 12–20 weeks of age. Animals were randomized based on age, weight, plasma Apob100 levels, HLA-A2, H2Db and H2Kb expression. Diet and water were available ad libitum. At sacrifice, mice were anesthetized by a subcutaneous injection (120 μl) of a cocktail containing ketamine (40 mg/ml), atropine (50 μg/ml) and sedazine (6.25 mg/ml). Subsequently, the mice were euthanized and exsanguinated by femoral artery transection and perfusion with PBS through the left cardiac ventricle.

### Immunization

Briefly, bone marrow was harvested from femurs and tibia from HHD mice. Bone marrow cells were cultured in complete IMDM (Lonza), IMDM supplemented with 8% FCS (GE Healthcare), 100 U/ml penicillin/streptomycin (Lonza), 2 mM Glutamax (Invitrogen) supplemented with 20 ng/ml GM-CSF (ImmunoTools) in non culture treated petri dishes at a concentration of 0.8*10^6^ cells/ml (10 ml per plate). After 3 days 10 ml fresh complete IMDM with 20 ng/ml GM-CSF was added to the petridishes. On day 6, 10 ml medium was carefully aspirated avoiding removal of cells, and replaced with complete IMDM with 20 ng/ml GM-CSF. At day 9 non/slightly adherent DCs were harvested and cultured overnight with 25 μg/ml high molecular weight poly(I:C) (Invivogen) in complete IMDM in non culture treated petri dishes. DCs were harvested and pulsed with peptides (10 μM) or vehicle (DMSO) for 2 hours at 37 °C in complete IMDM in 50 ml falcon tubes (10*10^6^ cells/ml) under constant agitation to avoid sticking to the plastic. DCs were washed twice with PBS and 2*10^6^ cells were intravenously infused through the lateral tail vein.

In the atherosclerosis studies mice received an i.v. booster vaccination consisting of PBS (control group), or PBS with 5% DMSO and 50 μg high molecular weight Poly(I:C) (VacciGrade™, Invivogen) and 50 μg αCD40 (FGK4.5, BioXcell) (vehicle group), or PBS with 5% DMSO and 30 μM peptide (approximately 67 μg/peptide) and 50 μg high molecular weight Poly(I:C) and 50 μg αCD40 (vehicle group), in a total volume of 200 μl, a week after DC vaccination.

### Primary cell preparation

For restimulation culture and flow cytometric analysis of lesional immune cells, aorta’s were isolated from just above the heart until at the height of the heart apex and cleaned from adipose tissue. Aorta’s were minced using scissors and digested in 0.5 ml PBS containing 400 U/ml collagenase type I (Sigma-Aldrich) and 120 U/ml collagenase type XI (Sigma-Aldrich) from *Clostridium histolyticum*, 60 U/ml type I-s hyaluronidase from bovine testes (Sigma-Aldrich) and 60 U/ml DNase 1 (Sigma-Aldrich) for 30 minutes at 37 °C under constant agitation. Aorta digests of 3 mice were pooled and mashed over 70μm strainers in RPMI to obtain single cell suspensions. Spleens and mediastinal lymph nodes were harvested and mashed over 70μm strainers to obtain single cell suspensions. At sacrifice, blood was obtained through terminal retro-orbital bleeding. Red blood cells from blood were lysed twice and splenocytes suspensions once, through incubation with 1 ml ACK lysis buffer (NH_4_Cl 150 mM, KHCO_3_ 10 mM, Na_2_EDTA 0.1 mM) for 30 seconds at room temperature.

### Evaluation of peptide specific immune responses

For measurement of antigen-specific CD8+ T cell responses, approximately 2.5*10^6^ splenocytes were incubated with indicated concentrations of individual peptides in U bottom plates. For assessment of specific CD8 T cells in mediastinal lymph nodes, approximately 1.5*10^6^ cells were incubated with 10 μM of all peptides combined in U bottom plates. For measurement of peptide specific CD8 T cell responses in aorta,cells from 3 aorta digests were combined, approximately 20*10^5^ viable CD45^+^ cells (flow cytometric analysis), and incubated with with 10 μM of all peptides combined in V-bottom plates. Cells were incubated with peptides for 4 h together with Brefeldin A (BD Biosciences) and Monensin (BD Biosciences) in RPMI (Lonza), supplemented with 8% FCS, 100 U/ml penicillin/streptomycin and 2 mM Glutamax.

### Flow cytometry

Extracellular staining of single cell suspensions was performed in PBS with 2% FCS and δCD16/32 antibody (93, Biolegend) and eBioscience™ Fixable Viability Dye eFluor™ 780 (ThermoFisher) to discriminate between living and dead cells at 4°C for 30 minutes. For intracellular cytokine staining, cells were incubated in Cytofix/Cytoperm™ Buffer (BD Biosciences) for 15 minutes at 4°C after extracellular staining, then washed twice with Perm/Wash Buffer (BD Biosciences) and stained in Perm/Wash Buffer for 45 minutes at 4°C. The following antibodies were purchased at BD biosciences: IL-17-FITC (TC11-18H10), F4/80-BV421 (BM8), CD4-V500 (RM-4-5), CD19PE-Cy7 (1D3). CD3 V500 (500A2). The following antibodies were purchased at Biolegend: CD8-BV510 (53-6.7), NK1.1-BV650 (PK136), CD4-PerCP (RM4-5), CD45-AF700 (30-F11), CD11b-PE/Dazzle 594 (M1/70), Thy1.2-PE-Cy7 (53-2.1), TNF-δ-PE (MP6-XT22), CD11c-FITC (N418), Ly6G-PerCP (1A8), Ly6C-APC (HK1.4). The following antibodies were purchases at ThermoFisher: IFN-Y-eFluor 450 (XMG1.2), IL-10-APC (JES5-16E3), CD11b –eVolve 605 (M1/70), MHCII-eVolve 655 (M5/114.15.2), CD19-PE (eBio1D3), CD8-PE-TR (5H10), CD62L-PerCP/Cy5.5 (MEL-14), CD44-APC (IM7). Compensation measurements were performed using UltraComp eBeads (ThermoFisher) and ArC Amine-Reactive Compensation Beads (ThermoFisher). Cells were analyzed with a Cytoflex S flow cytometer (Beckman Coulter) with Cytexpert 2.0 software (Beckman Coulter) and further analyzed using FlowJo software (Tree Star, inc.).

### Histology

Hearts were cut in half and incubated in OCT medium for 30 minutes. After 30 minutes hearts were fast frozen on dry ice, and stored at −80°C before cryosections (10 μm) of the aortic root were collected on Superfrost Plus™ Adhesion Microscope Slides (ThermoFisher) at 70 μm intervals (7 slides/mice). Neutral fats were stained with Oil Red O to assess lesion size in five subsequent sections of the heart within the three aortic valve area. Lesion collagen content was determined with Masson trichrome staining (Sigma-Aldrich). Corresponding sections analyzed for plaque area and collagen content were immunohistochemically stained for macrophages with MOMA-2 antibody (Sanbio, 1:1000 dilution), CD8 T cells with CD8 antibody (Ly-2, 1:100 dilution, BD Pharmingen). Slides were blocked with 5% milk powder before primary antibody was added for 2 h at RT, after which primary antibody was incubated overnight at 4°C. Endogenous peroxidase activity was blocked by incubating slides in 0.3% Hydrogen peroxide for 30 minutes at RT. Then slides were incubated for 1 h at RT with a polyclonal Rabbit Anti-Rat Ig HRP (DAKO), after which VECTASTAIN ABC HRP Kit (Vector Laboratories) was used. Stained with NovaRed (Vector Laboratories).

## Supplementary information

Induction of HLA-A2 restricted CD8 T cell responses against ApoB100 peptides does not affect atherosclerosis in a humanized mouse model.

## Data Availability

The data generated and analyzed during the current study are available from the corresponding author on reasonable request.
