# Supplementary material for: Induction of HLA-A2 restricted CD8 T cell responses against ApoB100 peptides does not affect atherosclerosis in a humanized mouse model
Source: Sci Rep. 2019 Nov 22;9:17391. doi: 10.1038/s41598-019-53642-z (PMC6874568; doi:10.1038/s41598-019-53642-z)
Supplement: Supplementary file 1 — Induction of HLA-A2 restricted CD8 T cell responses against ApoB100 peptides does not affect atherosclerosis in a humanized mouse model. [file 41598_2019_53642_MOESM1_ESM.pdf]

Title:

Induction of HLA-A2 restricted CD8 T cell responses against ApoB100 peptides does not affect atherosclerosis in a humanized mouse model.

Authors:

Frank H. Schaftenaar[1]*, Jacob Amersfoort[1], Hidde Douna[1], Mara J. Kröner[1], Amanda C. Foks[1], Ilze Bot[1], Bram A. Slütter[1], Gijs H.M. van Puijvelde[1], Jan W. Drijfhout[2], Johan Kuiper[1]*

Affiliations:

[1] Division of BioTherapeutics, Leiden Academic Centre for Drug Research, Leiden, The Netherlands

[2] Department of Immunohematology and Blood Transfusion, Leiden University Medical Center, Leiden, The Netherlands

* f.h.schaftenaar@lacdr.leidenuniv.nl

* j.kuiper@lacdr.leidenuniv.nl

Supplementary Fig. S1

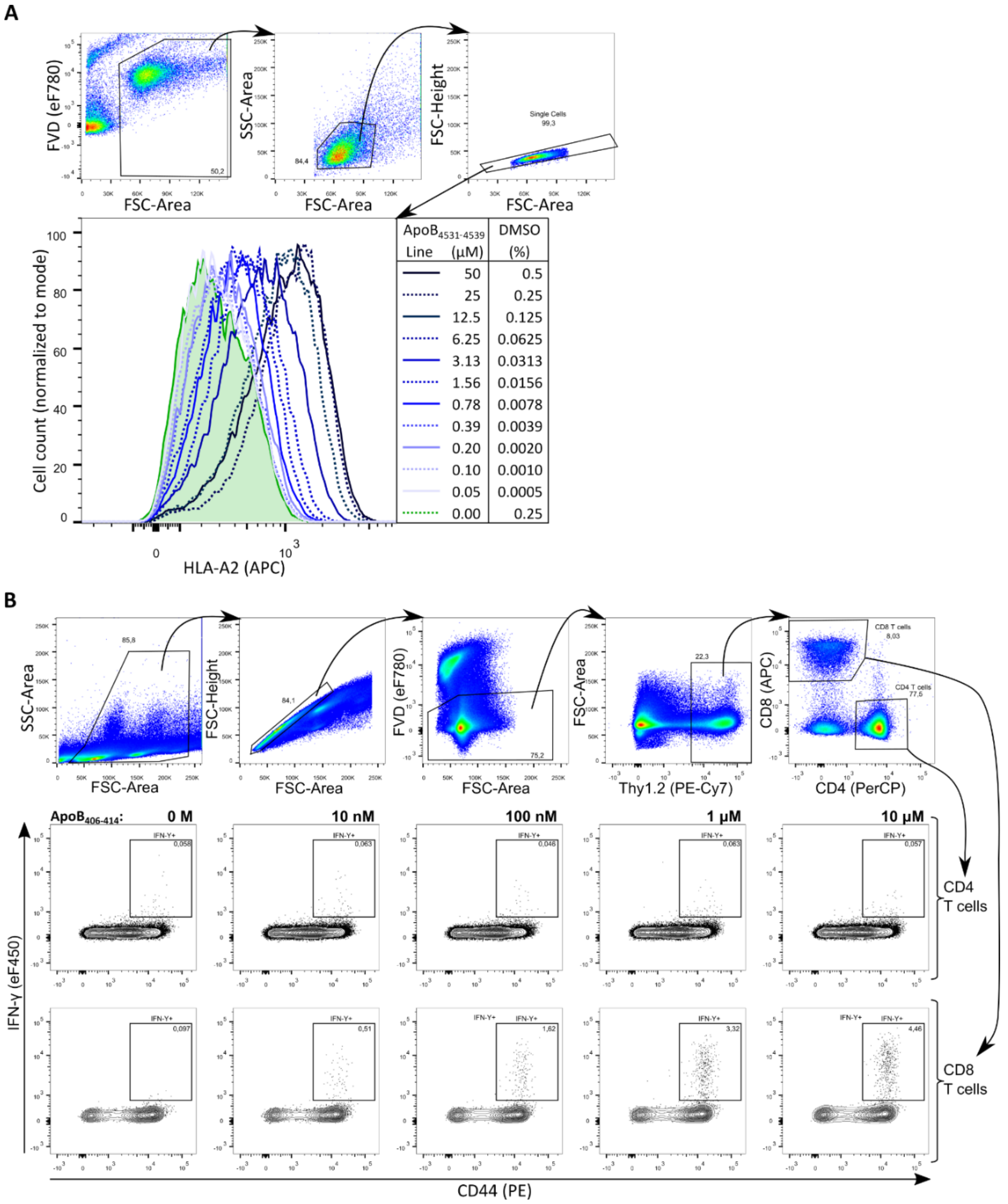

Supplementary Fig. S1: Gating strategy for T2 stabilization assay and ex vivo splenocyte peptide restimulation. (**A**) Representative flow cytometry plots of the T2 stabilization assay gating strategy, exemplified by $ApoB_{4531-4539}$ induced HLA-A2 stabilization on T2 cells. First living cells were gated based on FSC and fixable viability dye (FVD) staining. Then T2 cells were gated based on FSC and SSC after which singlets were gated based on FSC-A and FSC-H. The median fluorescent intensity in the APC channel in the living, singlet T2 cell population, was then determined. (**B**) Representative flow cytometry plots of the gating strategy of ex vivo CD4 and CD8 T cell stimulation as exemplified by $ApoB_{406-414}$ splenocyte stimulation. First small debris and large cell aggregates were gated out based on FSC-A and SSC-A. Next single cells were gated based on FSC-A and FSC-H, after which T cells were gated based on Thy1.2 expression. Subsequently CD4 and CD8 T cells were gated. Peptide specific T cell responses were assessed by gating for CD44 and IFN-γ double positive cells.

Supplementary Fig. S2

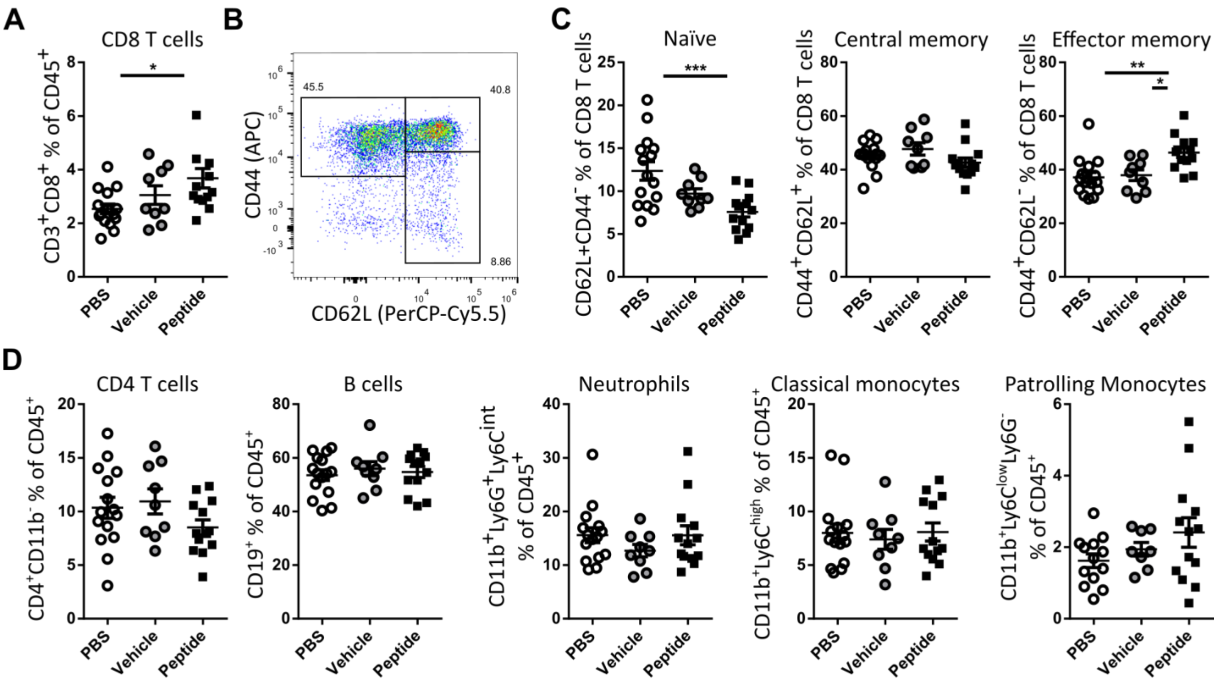

Supplementary Fig. S2: Peptide vaccination enhances effector memory CD8 T cells in circulation. (**A**) CD8 T cells were gated based on expression of CD3 and CD8 with flow cytometry and quantified. (**B**) Representative flow cytometry plot of the CD8 T cell population for the gating of $CD44^{+}CD62L^{-}$ effector memory CD8 T cells, $CD44^{+}CD62L^{+}$ central memory CD8 T cells, and $CD62L^{+}$CD44 naïve^{-} CD8 T cells. (**C**) Quantification of memory and naïve CD8 T cell populations. Statistical analysis was performed with 1-way ANOVA and Tukey's multiple comparisons test. Depicted as mean with SEM, * p < 0.05, ** *p* < 0.01, *** *p* < 0.001.

Supplementary Fig. S3

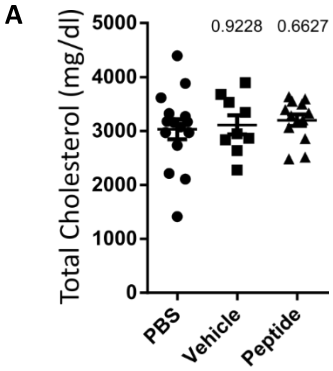

Supplementary Fig. S3: Total cholesterol levels are not affected by peptide vaccination. (**A**) Cholesterol levels in blood serum, obtained after sacrifice. Statistical analysis was performed with 1-way ANOVA and Tukey's multiple comparisons test. Depicted as mean with SEM, no significant data.

Supplementary Fig. S4

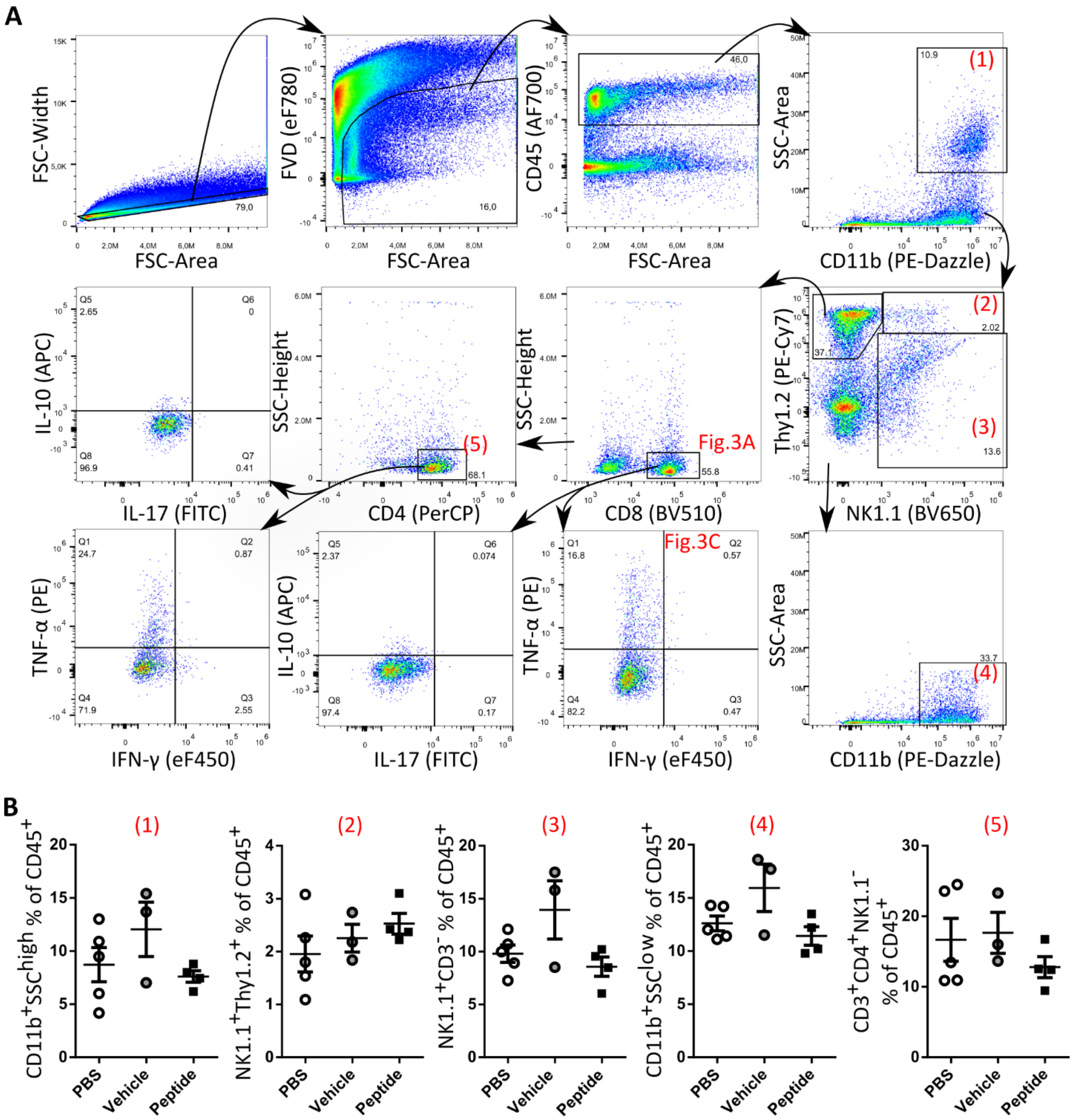

Supplementary Fig. S4: Gating strategy for aortic arch immune cell populations. (**A**) Representative flow cytometry plots of the gating of aortic cell populations. Cell populations in peptide stimulation cultures of mesenteric lymph nodes and spleen were gated similarly. Singlets were gated based on FSC-W and FSC-A. Viable cells were gate based on size (FSC-A) and low fluorescent viability dye (FVD) fluorescence. Immune cells were selected through gating on CD45^{+} cells. (1) A CD11b^{+}SSC^{high} was gated. From the CD11b^{+}SSC^{high} negative a Thy1.2^{+}NK1.1^{+} (2), a Thy1.2^{-}NK1.1^{+} (3) and Thy1.2^{+}NK1.1^{-} were gated. From the Thy1.2^{-}NK1.1^{-} population CD11b^{+} cells were gated (4), and from the Thy1.2^{+}NK1.1^{-} population CD8^{+}SSC^{low} cells were gated (CD8 T cells, Fig. 3A). From the CD8^{+}SSC^{low} negative population CD4^{+}SSC^{low} cells (5) were gated, being CD4 T cells. The CD4 T cell and CD8 T cell populations were then assessed for cytokine expression in IFN-γ vs TNF-α, and IL-17 vs IL-10 plots. (**B**) Quantifications of cell populations in cell cultures of the aortic arch as assessed by flow cytometry. Statistical analysis of B was performed with 1-way ANOVA and Tukey's multiple comparisons test, plotted as mean with SEM.

Supplementary Fig. S5

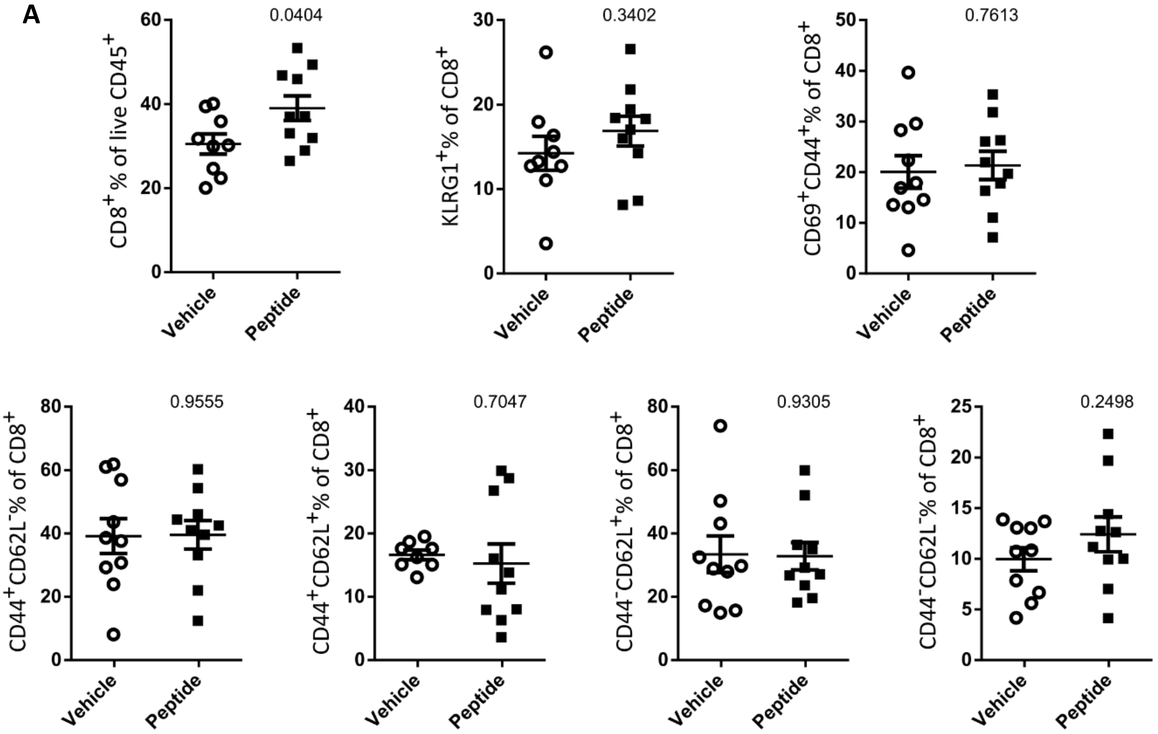

Supplementary Fig. S5: The aortic CD8 T cell phenotype is not altered by ApoB peptide vaccination (**A**) Quantification of the flow cytometry analysis of cells isolated from the aortic arch. Besides CD8 T cells, short lived effector cells (KLRG1$^+$), activated effector- (CD69$^+$CD44$^+$), effector memory- (CD44$^+$CD62L$^-$), central memory- (CD44$^+$CD62L$^+$), naïve- (CD44$^-$CD62L$^+$), and a second activated effector CD8 T cell population (CD44$^-$CD62L$^-$) were quantified. Statistical analysis was performed with T-tests. Depicted as mean with SEM, no significant data.

Supplementary Fig. S6

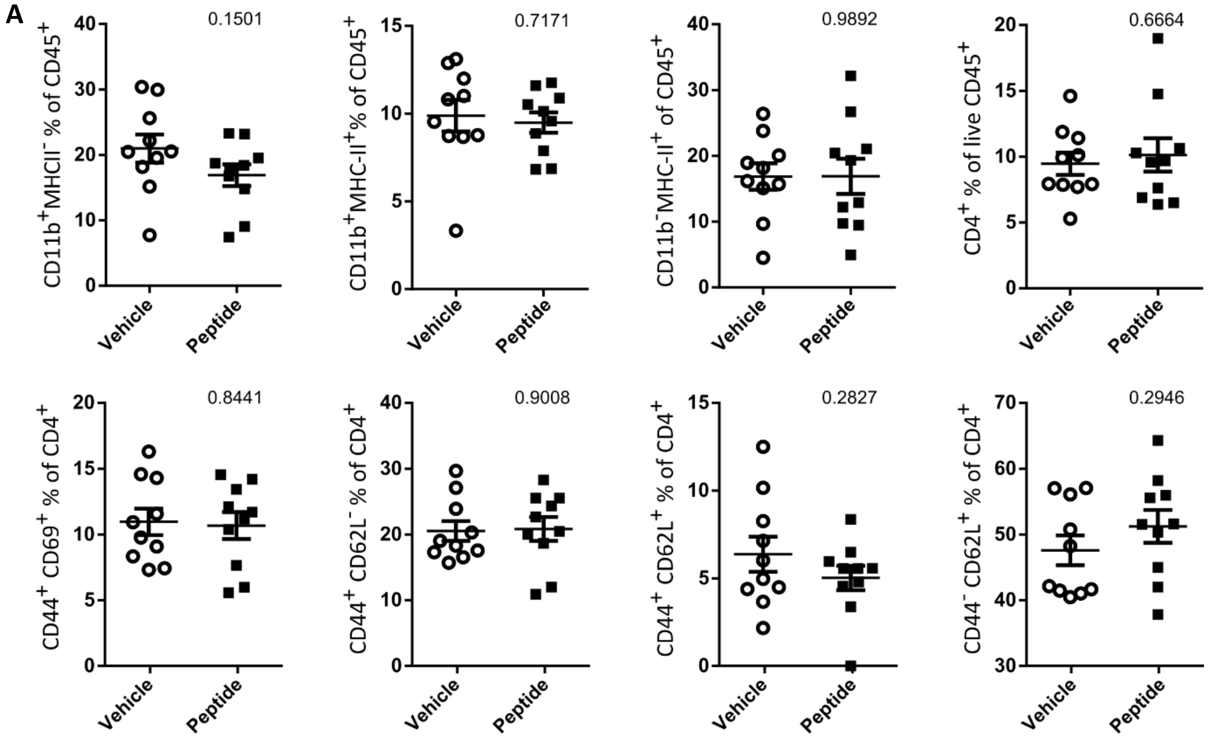

Supplementary Fig. S6: Vaccination with ApoB peptides did not significantly alter immune populations beside CD8 T cells. (**A**) Quantification of the flow cytometry analysis of cells isolated from the aortic arch of male HuBL-A2^{m+} mice. Statistical analysis was performed with T-tests. Depicted as mean with SEM, no significant data.

Supplementary Fig. S7

A

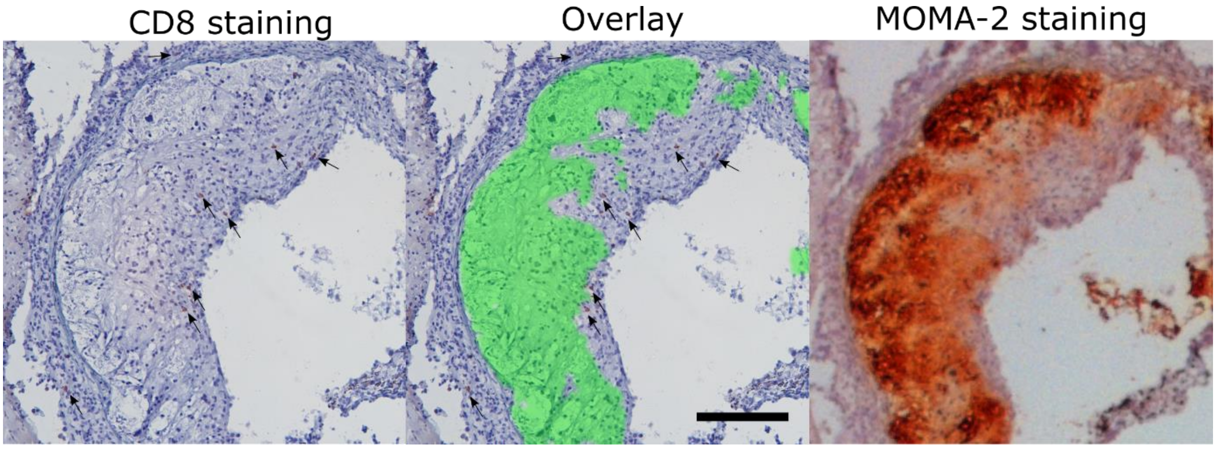

Supplementary Fig. S7: Co-localization of CD8 T cells with plaque macrophages is limited. (**A**) Representative micrographs of an ApoB-peptide treated HuBL-A2^{m-} mouse, immunohistochemically stained for CD8 (left panel) and MOMA-2 (right panel) in consecutive sections. The MOMA-2^{+} area (green in middle panel) was transferred to the original micrograph stained for CD8 to create an overlay (middle panel) to assess co-localization. Scale bar is 200 µm.
